# Development and Preliminary Evaluation of an AI-Assisted Clinical Decision Support System for Personalized Musculo-Skeletal Pain Management in Integrative Nursing Practice

**DOI:** 10.3390/healthcare14183027

**Published:** 2026-09-15

**Authors:** Şeyda Öztuna, Meryem Betos Koçak, Cihangir Işık

**Affiliations:** 1 Department of Psychiatric Nursing, Faculty of Health Sciences, Balıkesir University, 10145 Balıkesir, Türkiye; 2 Department of Family Medicine, Balıkesir Atatürk City Hospital, 10100 Balıkesir, Türkiye; mrym.abdullah@hotmail.com; 3 Forensic Medicine Polyclinic, Balıkesir Atatürk City Hospital, 10100 Balıkesir, Türkiye; dr.cihangirisik@yahoo.com

**Keywords:** artificial intelligence, biopsychosocial approach, clinical decision support systems, clinical skills education, digital health, integrative nursing, musculoskeletal pain, patient-centered care, respectful healthcare

## Abstract

**Highlights:**

**What are the main findings?**

- The machine learning models demonstrated good predictive performance, with 82.6% classification accuracy on the held-out retrospective test set, while the prototype was evaluated for preliminary workflow feasibility.
- The preliminary feasibility implementation showed favorable numerical trends in the AI-assisted group, without establishing clinical effectiveness or superiority over standard care.

**What are the implications of the main findings?**

- The prototype provides an initial framework for investigating AI-supported decision-making in integrative nursing practice, with potential applications in evidence-informed clinical decision-making, symptom monitoring, and continuity of patient-centered care.
- The integration of explainable AI and mobile decision-support technologies may contribute to more standardized, inclusive, and efficient healthcare delivery while preserving professional clinical judgment.

**Abstract:**

**Background/Objectives**: Musculoskeletal pain is a common condition that negatively affects quality of life and functional capacity. Integrative nursing emphasizes patient-centered and non-pharmacological approaches; however, variability in clinical decision-making persists due to reliance on clinician experience. This study aimed to design, retrospectively train, and evaluate the preliminary feasibility of a machine learning-based clinical decision support framework for personalized pain management within an integrative nursing approach in a Traditional and Complementary Medicine setting. **Methods**: This study included a retrospective cohort of 487 patients (mean age: 48.2 ± 14.7 years; 63.0% women) for machine learning model development and evaluation. The classification model predicted binary clinical improvement, defined as a ≥3-point reduction in VAS pain score, whereas the regression model predicted post-treatment VAS score. The resulting models were incorporated into the GETAIA prototype to generate modality-specific predicted post-treatment VAS scores and probabilities of clinically meaningful improvement for the three available T&CM modalities; these predicted profiles were compared to provide a model-derived treatment recommendation for clinical review. In addition, a prospective, non-randomized pilot involving 42 patients (21 AI-assisted and 21 standard care) was conducted to assess preliminary point-of-care workflow feasibility. **Results**: Significant reductions in pain intensity were observed across all treatment groups (*p* < 0.001). Clinically meaningful improvement rates were 76.2% for cupping therapy, 68.3% for acupuncture, and 58.8% for mesotherapy (*p* = 0.007). The prototype demonstrated 78.3% concordance with historical clinician treatment decisions, which was interpreted as an internal model-fidelity measure reflecting the reproduction of historical clinical decision patterns rather than evidence of improved decision-making, clinical effectiveness, or external validity. During a preliminary prospective feasibility implementation, clinically favorable numerical trends were observed in the AI-assisted group compared with standard care, including clinically meaningful improvement in 85.7% (18/21) versus 71.4% (15/21) of patients (*p* = 0.449) and mean VAS reductions of 3.9 ± 1.2 versus 3.2 ± 1.4 (*p* = 0.092). These exploratory differences were not statistically significant and were not intended to establish comparative clinical effectiveness. On the held-out test set, the primary XGBoost classification model achieved an accuracy of 82.6% and an AUC-ROC of 0.87 (95% CI: 0.82–0.92), while the regression model predicting post-treatment VAS achieved an MAE of 0.87. Key predictors of improvement included diagnosis, baseline pain intensity, and age. **Conclusions**: The findings support the preliminary development and evaluation of a machine learning-based decision-support framework and GETAIA prototype for individualized pain management within an integrative nursing context. The retrospective analysis demonstrated predictive performance of the evaluated models, while the small, non-randomized prospective pilot provided preliminary information on point-of-care workflow integration. These findings do not establish the clinical effectiveness, superiority, routine-care readiness, or external validity of a fully implemented clinical decision support system. Larger, prospective, multicenter studies are required to evaluate clinical effectiveness, generalizability, usability, and implementation readiness.

## 1. Introduction

Musculoskeletal pain, including low back, neck, and headache-related conditions, is a leading cause of disability and reduced quality of life worldwide, contributing substantially to healthcare utilization and functional limitations [1]. Effective management of these conditions requires not only symptom control but also a holistic, patient-centered approach addressing physical, psychological, and functional dimensions of health.

Within this context, integrative nursing has emerged as a comprehensive care model emphasizing evidence-based, non-pharmacological interventions, individualized care planning, and therapeutic relationships [2,3]. It integrates complementary approaches with conventional care to improve outcomes in chronic pain, where multimodal and interdisciplinary strategies are essential [4,5]. Chronic musculoskeletal pain is not only a physical condition but also a complex biopsychosocial phenomenon associated with psychological distress, anxiety, depressive symptoms, maladaptive coping strategies, and reduced quality of life. Recent evidence highlights that psychological and social determinants significantly influence pain persistence, treatment response, and functional outcomes in individuals with chronic musculoskeletal conditions [6]. Therefore, psychiatric nursing perspectives, including holistic assessment, therapeutic communication, psychosocial support, and patient empowerment, represent essential components of comprehensive and person-centered pain management. Theoretically, this approach is firmly anchored in Watson’s Theory of Human Caring and Kolcaba’s Comfort Theory. Watson’s framework emphasizes transpersonal caring, holistic assessment, and human dignity, which guide the integration of patient-centered values into clinical decision-making. Complementarily, Kolcaba’s Comfort Theory posits that addressing physical, psychospiritual, environmental, and sociocultural comfort enhances health-seeking behaviors and recovery. Together, these theoretical foundations underscore that digital and AI-driven innovations must enhance—rather than obscure—the relational, comfort-oriented, and holistic essence of clinical nursing practice [7,8].

Despite increasing adoption of integrative approaches, clinical decision-making in musculoskeletal pain remains highly variable and largely dependent on clinician experience. This variability contributes to inconsistencies in treatment selection and outcomes across healthcare settings [2]. In addition, chronic pain represents a complex biopsychosocial condition that cannot be adequately addressed through single-variable decision frameworks [9].

Recent advances in artificial intelligence (AI) and machine learning offer promising solutions by enabling data-driven prediction, treatment optimization, and clinical decision support. AI-based systems have demonstrated potential for supporting personalized care strategies and technology-assisted clinical decision-making across different healthcare contexts, although challenges related to clinical integration and implementation remain [10,11,12]. However, most existing applications remain focused on diagnostic or non-nursing domains, with limited integration into nursing-led or integrative care frameworks.

Integrating AI-based decision support systems into nursing practice may support evidence-informed clinical decision-making by facilitating individualized care planning and structured assessment. Such an approach is conceptually aligned with principles of inclusive and respectful healthcare; however, these dimensions were not directly evaluated in the present study [13].

While recent applications of AI in acupuncture and complementary medicine have explored specific computational tasks, such as acupuncture point selection, pattern recognition, and symptom classification, these applications have generally addressed individual components of clinical decision-making rather than providing an integrated nursing-centered framework for personalized musculoskeletal pain management [14]. Current literature lacks studies that combine integrative nursing principles (e.g., longitudinal symptom monitoring and patient satisfaction) with AI-driven personalized decision support across multiple Traditional and Complementary Medicine (T&CM) modalities, particularly those evaluating predictive performance alongside preliminary point-of-care feasibility and workflow integration.

Accordingly, this study aimed to develop and preliminarily evaluate a machine learning-based clinical decision-support framework and prototype (GETAIA) for personalized musculoskeletal pain management within an integrative nursing framework in a Traditional and Complementary Medicine setting, informed by patient-centered, inclusive, and respectful care principles as a theoretical and intended orientation.

## 2. Materials and Methods

### 2.1. Study Design

This study employed a quantitative, retrospective, descriptive, and correlational design.

### 2.2. Study Population and Sample

The study population consisted of all consecutive patients who attended the T&CM center between 10 October 2021 and 15 April 2024. To ensure that the sample accurately reflected routine, real-world clinical practice and to minimize selection bias, broad eligibility criteria were applied without arbitrary restrictions on age or sex.

Exclusion of patients was strictly limited to predefined clinical contraindications or missing primary outcomes. Specifically, the eligibility criteria were defined as follows:

Inclusion Criteria: (1) Patients aged 18–65 years presenting with chronic musculoskeletal pain conditions routinely managed in the T&CM center, including regional mechanical musculoskeletal pain disorders (e.g., low back pain, neck pain, and knee osteoarthritis-related pain) and selected chronic pain conditions such as fibromyalgia and headache/migraine-related musculoskeletal complaints; (2) baseline Visual Analog Scale (VAS) pain score ≥4; (3) patients scheduled to receive T&CM therapies (cupping therapy, acupuncture, or mesotherapy).

Exclusion Criteria: (1) Active local cutaneous infections or severe dermatological lesions at the application site; (2) Severe systemic diseases, active malignancy, bleeding disorders, primary inflammatory rheumatologic diseases, or neurological disorders with predominant neuropathic pain characteristics (e.g., diabetic neuropathy, severe radiculopathy, or peripheral nerve injury); (3) Pregnancy or lactation; (4) Previous T&CM treatment within the last 3 months; (5) Incomplete clinical evaluations or missing primary outcome data.

The final analyzed sample included 487 patients (307 women and 180 men) who met the predefined eligibility criteria and had available primary outcome data. Remaining missing covariate data were handled using Multiple Imputation by Chained Equations (MICE). By capturing consecutive admissions across a 2.5-year window, the sample preserves the natural heterogeneity of patients routinely seeking T&CM care, thereby enhancing external validity while mitigating selection and extraction bias.

To account for variability among musculoskeletal pain phenotypes, diagnostic categories were retained as predictive variables in the machine learning framework. This approach allowed the model to incorporate diagnosis-related differences in treatment response while reducing potential confounding arising from major differences in pain mechanisms.

### 2.3. Data Sources and Data Collection

Clinical data were retrospectively extracted from certified institutional Electronic Health Records (EHR), including anamnesis forms, clinical evaluations, and treatment records, by two trained clinical researchers (a senior physician and a specialist nurse) using a standardized digital abstraction protocol. Extracted variables encompassed demographic characteristics, clinical diagnoses, treatment modalities (cupping therapy, acupuncture, and mesotherapy), number of sessions, and Visual Analog Scale (VAS) scores. To assess inter-rater reliability, all 487 eligible patient records were independently reviewed by both researchers using the same standardized digital abstraction protocol. Agreement between the two reviewers was assessed using Cohen’s kappa for categorical variables and the intraclass correlation coefficient (ICC) for continuous measures. To minimize extraction bias, data abstractors were blinded to final patient treatment outcomes during initial record processing.

Over the 2.5-year data collection window, clinical definitions and diagnostic coding were maintained under standardized T&CM institutional guidelines to ensure data harmonization. Clinical diagnosis and treatment planning were routinely performed by certified T&CM physicians, while integrative nursing staff contributed to symptom monitoring, patient follow-up, supportive care, and continuity of care throughout the treatment process. Patient satisfaction was measured at the conclusion of treatment using a 5-point Likert scale questionnaire (1 = Very Dissatisfied to 5 = Very Satisfied).

All T&CM interventions were delivered according to standardized institutional clinical protocols. For acupuncture, needles were retained for approximately 20–30 min after insertion, with a total session duration of approximately 30–45 min, including patient preparation, skin disinfection, needle placement, and removal. During cupping therapy, cups remained in place for approximately 5–10 min, resulting in a total session duration of approximately 20–30 min, including patient preparation, sterilization, application, and post-procedure care. For mesotherapy, the active injection procedure lasted approximately 10–20 min depending on the treatment area, whereas the overall clinical session duration was approximately 30–45 min, including patient preparation, topical anesthesia when indicated, and post-procedure care. Session duration was standardized within each treatment modality according to institutional protocols and therefore was not treated as an independent analytical variable in the statistical analyses.

### 2.4. Data Processing

Data were extracted into structured Excel files and processed over a three-month period. Data cleaning and preprocessing included manual review and automated validation. The final dataset was converted into CSV format for machine learning analysis. For clinical integration, data were securely connected to the GETAIA mobile application via API architecture, ensuring data security and confidentiality.

The AI-assisted decision-support prototype (GETAIA) integrates supervised machine learning models with a rule-based clinical interpretation layer developed using clinical T&CM records. For point-of-care recommendation generation, GETAIA uses patient information available at baseline (T0), before treatment was finalized, including age, sex, baseline VAS pain score, symptom duration, comorbidity status, and diagnostic category. Actual treatment modality and number of treatment sessions were not used as baseline inputs for generating the treatment recommendation; these variables were available only from the retrospective clinical records and were used in retrospective analyses of observed treatment outcomes. The primary classification task estimated the probability of binary clinical improvement, defined as a ≥3-point reduction in post-treatment VAS score relative to baseline. A separate regression task estimated the post-treatment VAS score as a continuous outcome.

For treatment recommendation, the prototype generated modality-specific predicted outcomes for each of the three available T&CM modalities (cupping therapy, acupuncture, and mesotherapy) using the patient’s baseline characteristics. For each modality, the system generated a predicted post-treatment VAS score and a predicted probability of clinically meaningful improvement. These modality-specific predicted outcome profiles were compared within the rule-based clinical interpretation layer, and the modality with the most favorable overall predicted outcome profile, characterized by a lower predicted post-treatment VAS score and a higher predicted probability of clinically meaningful improvement, was presented as a model-derived T&CM recommendation for clinical review. The recommendation was not considered an “optimal” or clinically validated treatment selection; rather, it represented supportive decision-making information derived from patterns learned from the retrospective clinical dataset. The clinician reviewed these outputs alongside the routine clinical assessment and retained full responsibility for the final treatment decision.

#### Prototype Architecture and GETAIA Mobile Application Integration

To facilitate point-of-care usability, the machine learning models were integrated into a functional decision support prototype via the GETAIA mobile application. The system architecture operates on a structured, four-stage workflow designed to support nursing and clinical assessment:

- Data Input (Baseline Assessment/T0): During the initial clinical assessment, patient-specific predictor variables used by the trained models-including age, sex, baseline VAS pain score, symptom duration, comorbidity status, and diagnostic category-are entered into the GETAIA mobile application. At baseline (T0), before treatment selection, the GETAIA recommendation module uses only information available from the initial clinical assessment, including age, sex, baseline VAS pain score, symptom duration, comorbidity status, and diagnostic category. Actual treatment modality and number of treatment sessions are recorded subsequently and are not used as inputs for the baseline treatment recommendation.
- Model Processing: The baseline parameters are securely transmitted via Application Programming Interface (API) architecture to the cloud-hosted machine learning backend. The trained models process these baseline characteristics to generate predicted post-treatment VAS and the probability of clinically meaningful improvement for the available T&CM modalities. These predicted outcome profiles are compared across cupping therapy, acupuncture, and mesotherapy, and the modality associated with the most favorable predicted outcome profile is presented as a model-derived T&CM recommendation for clinical review.
- Recommendation Output (Point-of-Care Support): The system generates real-time decision-support outputs consisting of (i) a model-derived T&CM modality recommendation for clinical review (cupping therapy, acupuncture, or mesotherapy), (ii) the corresponding predicted post-treatment VAS score, and (iii) the predicted probability of achieving clinically meaningful improvement. The recommendation reflects the modality with the most favorable overall predicted outcome profile, based on the combination of a lower predicted post-treatment VAS score and a higher predicted probability of clinically meaningful improvement. The output is presented as supportive information rather than as a clinically validated optimal treatment selection.
- Human-in-the-Loop Governance: The model-derived recommendation served strictly as supportive clinical guidance to complement professional clinical reasoning. Final diagnostic and therapeutic decisions remained entirely under the responsibility of the healthcare professional, preserving personalized, patient-centered care. Actual post-treatment VAS scores and patient satisfaction Likert scores were subsequently collected post-intervention (T1) as clinical validation benchmarks.

### 2.5. Variables, Data Preprocessing, and Statistical Analysis

For baseline decision-support prediction, candidate predictors available at T0 included age, sex, baseline VAS pain score, symptom duration, comorbidity status, and diagnostic category. Treatment modality and number of treatment sessions were analyzed as retrospective treatment-related variables when evaluating observed treatment outcomes and were not available as inputs to the baseline recommendation module. These variables were selected based on clinical relevance established in prior T&CM literature and expert consensus in integrative nursing and pain management. Variables with extreme missingness (>20%) or negligible analytical value were excluded. For remaining features, the overall proportion of missing data was minimal (<3.5% across all variables, with exact missingness per variable being baseline VAS score 1.2%, symptom duration 2.8%, and comorbidity status 3.1%). Missing data were addressed using Multiple Imputation by Chained Equations (MICE). Categorical variables—including diagnostic categories and T&CM treatment modalities (cupping therapy, acupuncture, and mesotherapy)—were processed using One-Hot Encoding to avoid imparting artificial ordinal relationships to nominal clinical categories. Continuous variables were standardized using Z-score normalization. Given the compact and clinically targeted set of core predictor variables, dimensionality reduction techniques (such as Principal Component Analysis) were deliberately not applied to preserve the direct clinical interpretability and feature importance transparency of all input variables. To address class imbalance across diagnostic subgroups, the Synthetic Minority Over-sampling Technique (SMOTE) was applied strictly within training folds during cross-validation to eliminate data leakage.

Descriptive statistics were presented as mean ± standard deviation (SD) or median (interquartile range [IQR]) for continuous variables, and as frequencies and percentages (*n*, %) for categorical variables. Normality of continuous data was evaluated using the Shapiro–Wilk test and Q-Q plots. Between-group comparisons were performed using independent two-sample *t*-tests, Mann–Whitney U tests, one-way ANOVA, or Kruskal–Wallis tests, as appropriate. Categorical variables and agreement between AI recommendations and clinician decisions were evaluated using Pearson’s Chi-square test or Fisher’s exact test. Paired-sample *t*-tests or Wilcoxon signed-rank tests were applied to compare pre- and post-treatment VAS pain scores within each group.

To identify independent predictors of treatment success (defined as a ≥3-point reduction in VAS score), univariate and multivariate logistic regression analyses were conducted to report Odds Ratios (ORs) with 95% Confidence Intervals (CIs), adjusted for age, sex, baseline pain score, and diagnostic category. The correlation between pain reduction and patient satisfaction Likert scores was evaluated using Pearson’s or Spearman’s correlation coefficients (r). Machine learning model performance was evaluated using classification accuracy, precision, recall, and F1-score, while regression models were evaluated using Mean Absolute Error (MAE). Feature importance analysis was performed to quantify variable contributions. All statistical analyses were two-tailed, with statistical significance set at *p* < 0.05. Computations were performed using IBM SPSS Statistics (Version 28.0; IBM Corp., Armonk, NY, USA), Python 3.10 machine learning libraries (scikit-learn), and Orange Data Mining (v3.39.0).

### 2.6. Sample Size Adequacy and Dataset Split

A formal prospective power analysis was not conducted due to the retrospective observational nature of the dataset. However, sample size adequacy for machine learning modeling was strictly evaluated based on established feature-to-sample ratio guidelines (minimum 10 to 15 events per candidate predictor variable) and cross-validation integrity. With 487 patient records and a focused set of clinically relevant predictor variables, the dataset provided a substantial sample relative to the dimensionality of the modeling problem. Furthermore, repeated 10-fold cross-validation and regularized model architectures, including XGBoost with L2 regularization and early stopping, were used to mitigate overfitting and enhance model stability within this sample.

The complete dataset (*n* = 487) was randomly partitioned into a training set (80%, *n* = 390) and an independent holdout test set (20%, *n* = 97) using stratified sampling to preserve baseline class distribution across pain severity and diagnostic categories. The training set was used for model optimization and internal cross-validation, while the test set was strictly reserved for final performance evaluation.

### 2.7. AI Model Development

Model development and evaluation were conducted in two sequential stages. First, the 80% training dataset (*n* = 390) was used for model development, including hyperparameter optimization and internal validation through stratified repeated 10-fold cross-validation (5 repetitions; 50 validation folds). All preprocessing, SMOTE resampling, and hyperparameter optimization procedures were performed within the training folds to minimize data leakage. Second, after completion of model development and selection of the optimal algorithm and hyperparameters, the finalized model was evaluated once on the independent 20% holdout test set (*n* = 97), which had not been used during model training, hyperparameter optimization, or cross-validation. Thus, cross-validation results represent internal model-development performance, whereas holdout test results represent the final out-of-sample evaluation of the finalized model.

Five distinct machine learning algorithms—Logistic Regression, Random Forest, Support Vector Machines (SVM), Gradient Boosting, and XGBoost—were systematically evaluated for predictive classification tasks. The primary target variable was binary clinical improvement, operationalized as a ≥3-point reduction in VAS score from baseline to post-treatment assessment. In the original cohort (*n* = 487), the outcome class distribution was moderately imbalanced (70.0% improved vs. 30.0% non-improved). To obtain more stable internal performance estimates and reduce reliance on a single training split, model training and internal validation utilized a Stratified Repeated 10-Fold Cross-Validation scheme (5 repetitions, 50 total folds) applied to 80% of the dataset (*n* = 390). Hyperparameter optimization via GridSearch CV and Synthetic Minority Over-sampling Technique (SMOTE) was executed strictly inside the training folds of each loop to eliminate data leakage. Model performance across folds is reported as Mean ± Standard Deviation along with 95% Confidence Intervals (95% CI). Finally, a completely unseen 20% holdout test set (*n* = 97) was preserved in its natural distribution for final out-of-sample evaluation. Hyperparameter optimization was systematically performed for all candidate algorithms across internal cross-validation folds, yielding the following search space and finalized optimal parameters:

- Logistic Regression: Evaluated penalty types (L1, L2) and inverse regularization strength C [0.001, 0.01, 0.1, 1.0, 10.0, 100.0]; optimal: L2 penalty with C = 1.0.
- Random Forest: Evaluated n_estimators [100, 200, 300, 500], max_depth [None, 5, 10, 15], and min_samples_split [2, 5, 10]; optimal: n_estimators = 200, max_depth = 10, min_samples_split = 5.
- Support Vector Machines (SVM): Evaluated kernels (linear, rbf) and regularization parameter C [0.1, 1.0, 10.0] with gamma [“scale”, “auto”]; optimal: rbf kernel, C = 1.0, gamma = “scale”.
- Gradient Boosting: Evaluated learning_rate [0.01, 0.05, 0.1], n_estimators [100, 200, 300], and max_depth [3, 5, 8]; optimal: learning_rate = 0.05, n_estimators = 200, max_depth = 5.
- XGBoost (Primary Model): Evaluated learning_rate (eta) [0.01, 0.05, 0.1], max_depth [3, 6, 9], n_estimators [100, 300, 500], and subsampling ratio [0.6, 0.8, 1.0]; optimal: max_depth = 6, eta = 0.05, n_estimators = 300, subsample = 0.8, and reg_lambda = 1.0.

Early stopping criteria (stopping after 20 rounds of stagnant validation loss) and L2 regularization were strictly implemented during XGBoost iteration to prevent overfitting. Among all candidate algorithms, XGBoost was identified as the optimal primary model based on its superior cross-validation performance (highest Mean Area Under the ROC Curve [AUC-ROC]).

Model evaluation was comprehensively expanded beyond standard metrics. Performance was quantified using Accuracy, Precision (Positive Predictive Value), Recall (Sensitivity), Specificity, F1-Score, and Area Under the Receiver Operating Characteristic curve (AUC-ROC), with 95% Confidence Intervals (95% CI) estimated across cross-validation folds. Model calibration was evaluated using the Brier Score and probability calibration curves to assess the agreement between predicted probabilities and observed clinical improvement. Additionally, confusion matrices were generated for holdout test evaluations to detail true positive, false positive, true negative, and false negative distributions.

A separate regression model was developed to estimate post-treatment VAS as a continuous outcome. The regression task was evaluated using mean absolute error (MAE), with lower values indicating smaller prediction errors.

The classification and regression models constituted the outcome-prediction component of the GETAIA architecture. For each available T&CM modality, the models generated a predicted post-treatment VAS score and an estimated probability of clinically meaningful improvement (defined as a ≥3-point reduction in VAS) using the patient’s baseline characteristics. These modality-specific predicted outcome profiles were then evaluated within the prototype’s rule-based clinical interpretation layer. The recommendation rule prioritized the modality associated with the most favorable overall predicted outcome profile, defined by a lower predicted post-treatment VAS score together with a higher predicted probability of clinically meaningful improvement. The modality with the most favorable profile across these two predicted outcomes was presented as the model-derived T&CM recommendation for clinical review. Thus, outcome prediction and treatment recommendation represented linked but analytically distinct components of the prototype. The recommendation was intended to provide structured decision-support information rather than to identify a clinically validated optimal treatment, and the final treatment decision remained the responsibility of the treating clinician.

### 2.8. Model Evaluation

The primary target outcome for classification was binary clinical success, explicitly operationalized as a clinically meaningful pain reduction defined by a ≥3-point reduction decrease in post-treatment Visual Analog Scale (VAS) score relative to baseline assessment. This threshold aligns with established consensus guidelines (IMMPACT criteria) and empirical clinical literature validating that a ≥2.5 to 3.0 point reduction on a 10-point scale corresponds to substantial clinical improvement and high patient satisfaction across diverse musculoskeletal diagnoses [15,16]. Patients meeting or exceeding this threshold were categorized as treatment responders, whereas those achieving less than a 3-point reduction were classified as non-responders.

Model performance was comprehensively evaluated on the completely unseen 20% holdout test set using the Area Under the Receiver Operating Characteristic Curve (AUC-ROC), Sensitivity (Recall), Specificity, Precision, Accuracy, and F1-score. To evaluate probability calibration, Brier scores and calibration curve slopes were calculated. To ensure robust statistical reporting, 95% confidence intervals (CI) for all performance metrics were derived using 1000 bootstrap resamples on the test set.

To ensure model transparency and clinical interpretability, feature importance was quantified using SHAP (SHapley Additive exPlanations) values based on cooperative game theory. SHAP values calculate the marginal contribution of each clinical feature to the output prediction across all permutation subsets. Global feature impacts were derived by averaging the absolute SHAP values across all test instances and normalizing them to relative percentage contributions. Methodology and reporting strictly adhere to the TRIPOD (Transparent Reporting of a multivariable prediction model for Individual Prognosis or Diagnosis) statement guidelines.

### 2.9. Prospective Pilot Feasibility and Point-of-Care Workflow Evaluation

To evaluate the real-time operational feasibility and point-of-care workflow integration of the framework, a prospective pilot observation was conducted with a cohort of 42 consecutive patients at the T&CM center. This pilot phase was designed primarily as a feasibility study rather than a fully powered clinical trial. The primary purpose was to assess point-of-care workflow integration and operational feasibility; comparative clinical outcomes were considered exploratory and were not intended to provide confirmatory evidence of treatment effectiveness. Patients were allocated via a 1:1 sequential pragmatic scheme into either the AI-assisted prototype group (*n* = 21) or the standard care group (*n* = 21) based on point-of-care clinical availability without formal randomization. Baseline comparability between the two pilot arms was formally evaluated, showing no statistically significant differences in mean age (47.1 ± 13.2 vs. 49.3 ± 14.1 years; *p* = 0.605), female distribution (61.9% vs. 66.7%; *p* = 0.749), or baseline VAS pain intensity (7.4 ± 1.5 vs. 7.2 ± 1.6; *p* = 0.681), with no statistically significant differences observed between the two pilot groups in these baseline characteristics.

- Standard Care: Defined as routine clinical management where physicians and nurses applied conventional clinical protocols and judgment without access to decision-support software.
- AI-assisted prototype: Defined as management where nurses and clinicians utilized the GETAIA mobile interface at the point of care, reviewing the AI-generated treatment recommendations and predicted success probabilities alongside standard assessment prior to finalizing treatment decisions.

Given the exploratory nature of this pilot evaluation, sample size (*n* = 42) was determined based on pilot feasibility guidelines (recommending 12–30 participants per arm) rather than a formal power calculation for statistical superiority. To address potential baseline imbalances in pain severity-specifically the higher baseline VAS scores observed in certain treatment subgroups (e.g., cupping therapy)-and to control for potential regression to the mean, group comparisons of pain reduction magnitude were conducted using Analysis of Covariance (ANCOVA) and multivariable regression modeling with baseline VAS score included as a continuous covariate.

### 2.10. Ethical Considerations

The study was conducted in accordance with the ethical principles outlined in the Declaration of Helsinki. Ethical approval was granted by the Institutional Ethics Committee (Decision/Protocol No: 2025/01/6, dated January 2025). The institutional ethics approval authorized both the retrospective analysis of electronic health records collected between October 2021 and April 2024 (*n*= 487) for model development and validation and the prospective pragmatic pilot evaluation (*n* = 42).

Informed consent was obtained from all participants involved in the study. For the retrospective component, informed consent was obtained in accordance with the procedures approved by the Institutional Ethics Committee. For the prospective pilot component, conducted between September 2025 and November 2025, written informed consent was obtained from all participants prior to enrollment. All patient identifiers were strictly anonymized to preserve confidentiality and maintain compliance with institutional and international data protection standards.

## 3. Results

### 3.1. Demographic and Clinical Characteristics

The study included 487 patients (307 females, 180 males), with a mean age of 48.2 ± 14.7 years. The distribution of treatment modalities was as follows: acupuncture (*n* = 205, 42.1%), cupping therapy (*n* = 168, 34.5%), and mesotherapy (*n* = 114, 23.4%). Detailed sociodemographic and clinical characteristics are presented in Table 1. For data extraction reliability, all 487 eligible patient records were independently reviewed by both researchers using the standardized digital abstraction protocol. Inter-rater agreement was high, with a Cohen’s kappa of 0.88 for categorical variables and an intraclass correlation coefficient (ICC) of 0.92 for continuous measures.

### 3.2. Treatment Outcomes

All treatment groups demonstrated statistically significant reductions in pain intensity following intervention (*p* < 0.001). Baseline pain scores were highest in the cupping therapy group (7.6 ± 1.4). Post-treatment pain scores were comparable across groups (cupping: 3.8 ± 1.9; acupuncture: 4.0 ± 1.8; mesotherapy: 4.0 ± 2.0), with no statistically significant between-group differences (*p* = 0.568).

**Figure 1:**
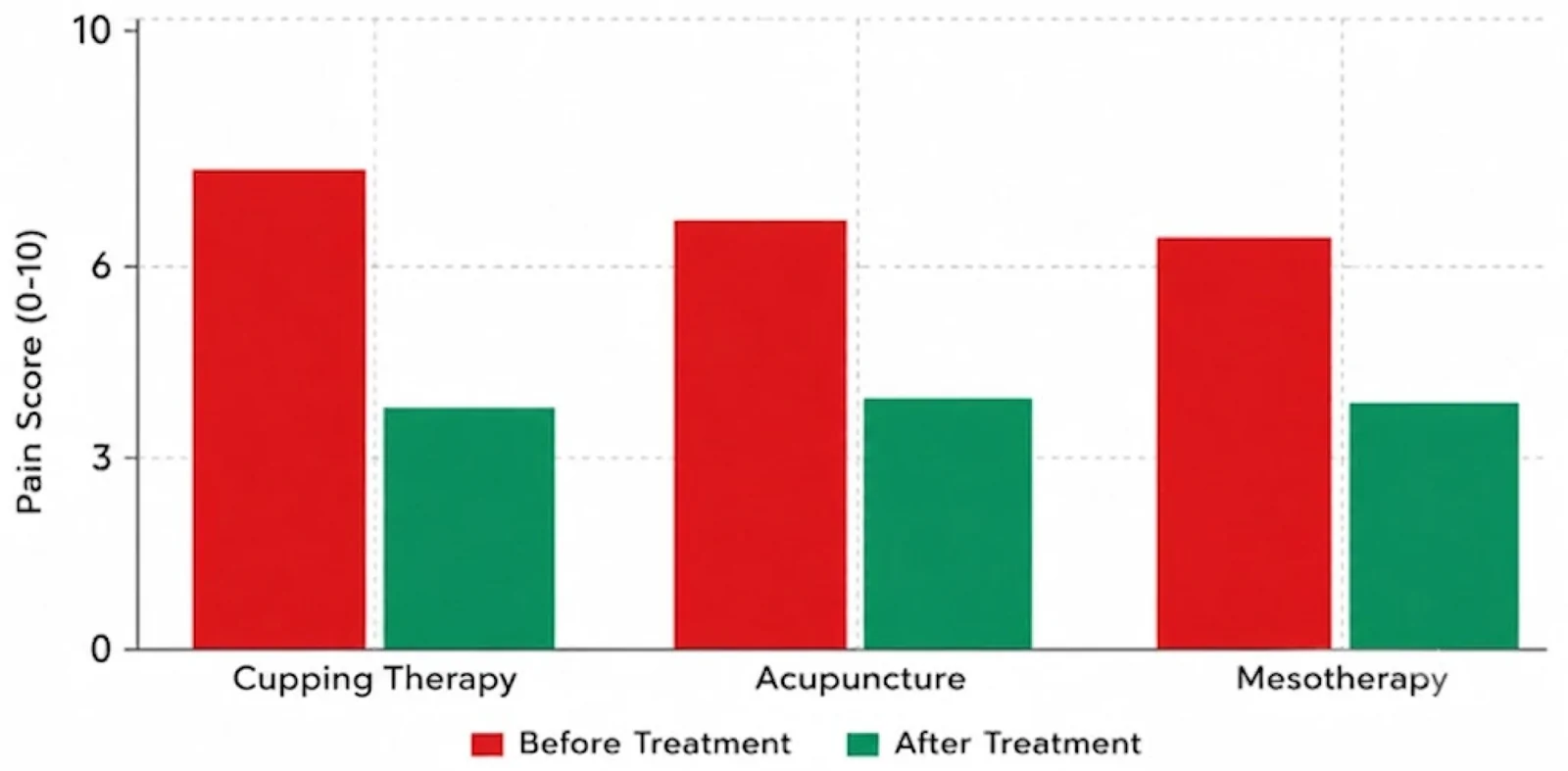
Pre- and Post-Treatment VAS Scores.

However, the magnitude of pain reduction differed significantly among interventions (*p* = 0.002), with the largest observed mean reduction in the cupping therapy group (3.8 ± 1.3; Cohen’s d = 1.05, 95% CI: 0.82–1.28), followed by acupuncture (3.2 ± 1.1; Cohen’s d = 0.82, 95% CI: 0.61–1.03) and mesotherapy (2.9 ± 1.4; Cohen’s d = 0.74, 95% CI: 0.51–0.97) (Figure 1). All intervention groups showed large to very large within-group effect sizes for pain reduction.

### 3.3. Subgroup Analysis by Diagnosis

Treatment response varied across diagnostic categories. Cupping therapy showed more favorable observed outcomes in low back and neck pain, whereas acupuncture yielded higher observed outcome values in headache/migraine and fibromyalgia. Mesotherapy demonstrated more favorable observed outcomes in knee pain cases (*p* = 0.278).

### 3.4. Clinically Meaningful Improvement

A clinically meaningful improvement (≥3-point reduction in VAS) was observed in 76.2% of patients in the cupping group, 68.3% in the acupuncture group, and 58.8% in the mesotherapy group (*p* = 0.007). After adjustment for age, sex, and diagnosis, cupping therapy was associated with higher odds of clinical improvement (OR = 1.68; 95% CI: 1.12–2.54; *p* = 0.012).

### 3.5. Predictors of Treatment Success

**Figure 2:**
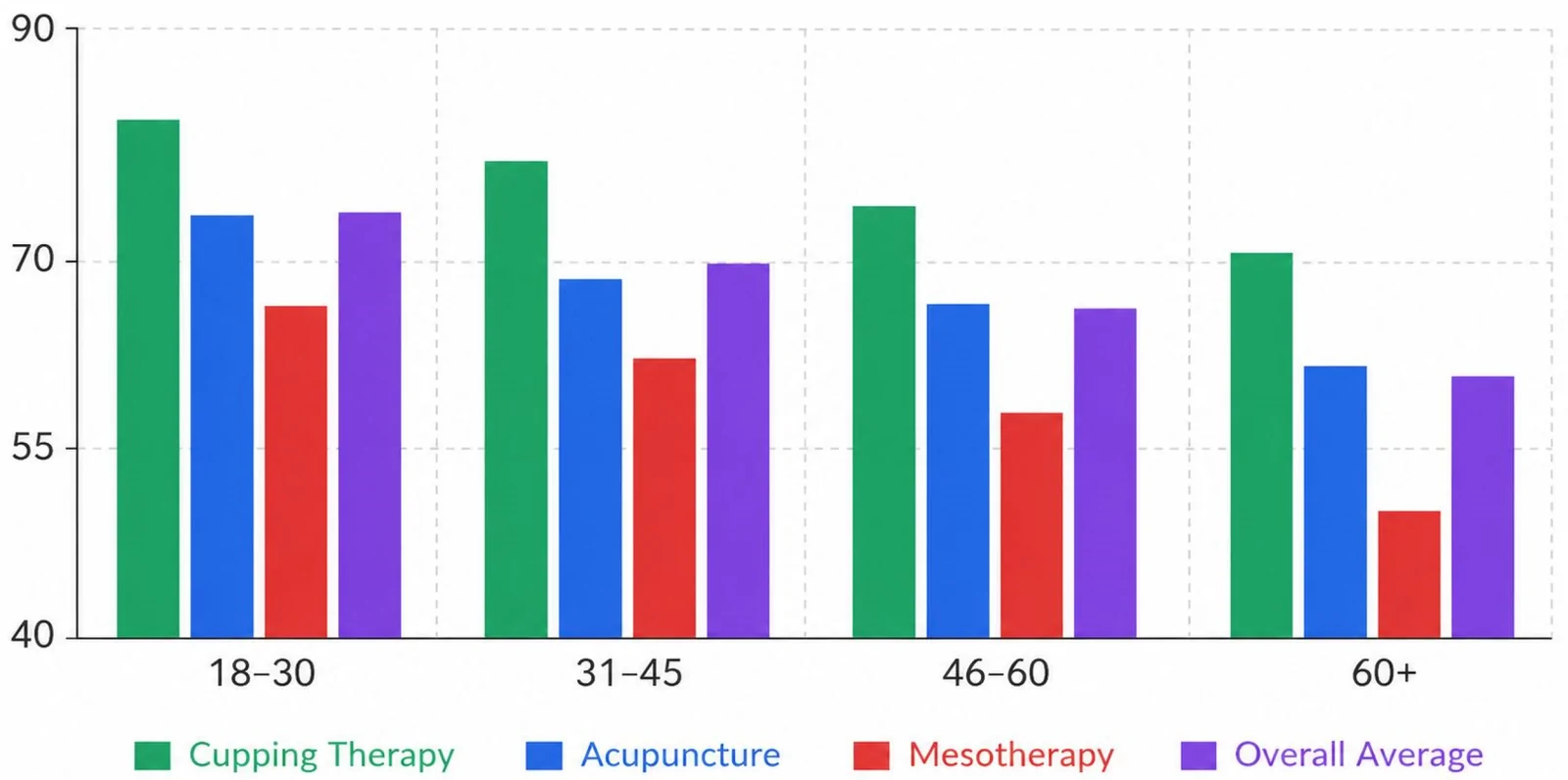
Treatment Success Rates by Age Groups.

Multivariate logistic regression analysis identified several independent predictors of clinical improvement. Diagnostic category was a strong determinant of outcome, with low back pain (OR = 2.14; 95% CI: 1.42–3.21; *p* < 0.001) and migraine (OR = 1.92; 95% CI: 1.25–2.98; *p* = 0.003) associated with increased odds of improvement. Higher baseline pain intensity (VAS ≥ 7) was associated with greater clinical improvement (OR = 1.89; 95% CI: 1.26–2.84; *p* = 0.002). Patients receiving ≥7 treatment sessions had a significantly higher likelihood of clinical improvement than those receiving 1–2 sessions (OR = 2.34; 95% CI: 1.58–3.47; *p* < 0.001). Patients aged ≤50 years demonstrated higher success rates compared with older patients (OR = 1.75; 95% CI: 1.18–2.59; *p* = 0.005) (Figure 2).

**Figure 3:**
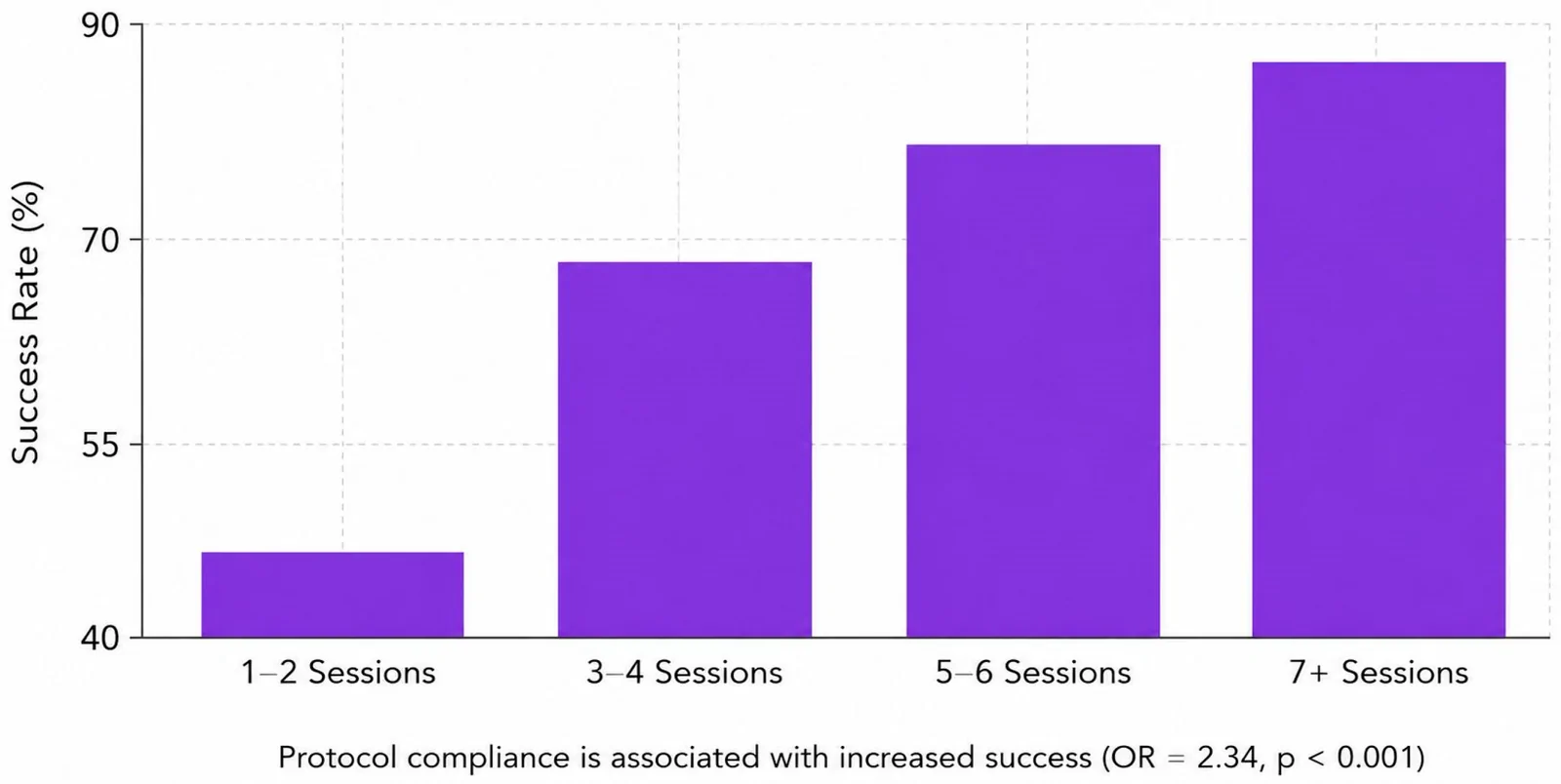
Success Rates According to Number of Treatment Sessions.

Treatment session frequency was also associated with clinical improvement, with patients receiving ≥7 treatment sessions achieving success rates exceeding 85%, compared with approximately 45% among those receiving 1–2 sessions (*p* < 0.001) (Figure 3).

### 3.6. Machine Learning Model Performance

**Figure 4:**
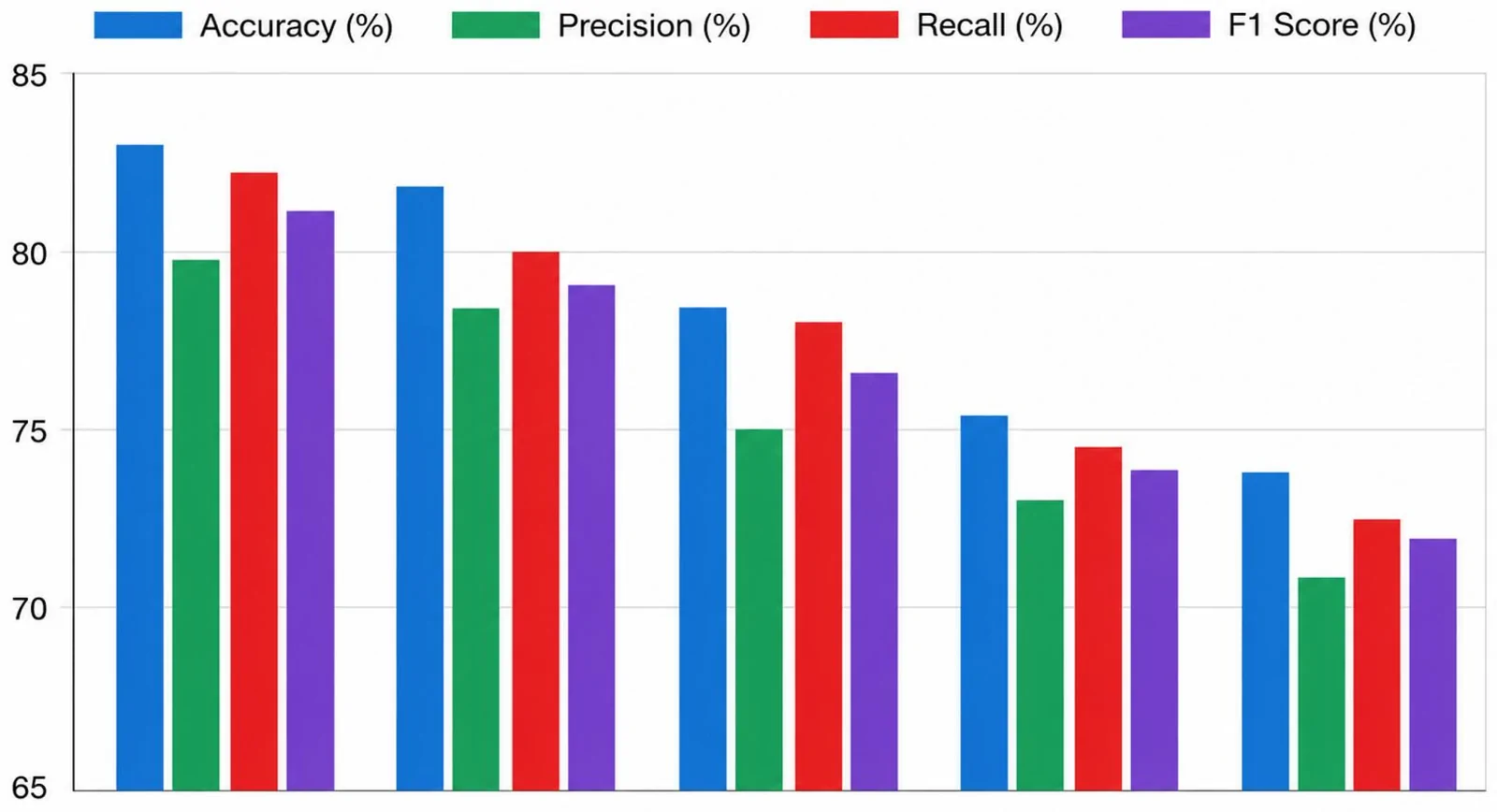
Performance Comparison of Classification Algorithms.

Model performance was evaluated across candidate algorithms using Stratified Repeated 10-Fold Cross-Validation (5 repetitions, 50 total folds) on the training set (*n* = 390). Among all evaluated algorithms, XGBoost achieved the highest predictive performance with a mean AUC-ROC of 0.88 ± 0.03 (95% CI: 0.85–0.91), outperforming Gradient Boosting (0.85 ± 0.03; 95% CI: 0.82–0.88), Random Forest (0.83 ± 0.03; 95% CI: 0.80–0.86), Support Vector Machines (0.79 ± 0.04; 95% CI: 0.75–0.83), and Logistic Regression (0.76 ± 0.04; 95% CI: 0.72–0.80) (Figure 4).

On the held-out test dataset (*n* = 97), the primary XGBoost classification model demonstrated good out-of-sample predictive performance for binary clinical improvement (≥3-point VAS reduction). The model achieved an AUC-ROC of 0.87 (95% CI: 0.82–0.92), with an accuracy of 82.6%. Based on the confusion matrix, sensitivity (recall) was 91.7%, specificity was 67.6%, precision was 82.1%, and the F1-score was 86.6%.

The confusion matrix for the holdout test set (*n* = 97) revealed 55 True Positives, 25 True Negatives, 12 False Positives, and 5 False Negatives. Model calibration analysis indicated excellent concordance between predicted probabilities and observed outcomes, yielding a Brier Score of 0.12 (where lower values reflect superior calibration) and a calibration curve slope near unity (0.96).

### 3.7. Regression Performance

The regression model demonstrated good predictive performance in estimating post-treatment VAS scores, with an overall mean absolute error (MAE) of 0.87. Subgroup analysis revealed lower prediction errors in low back pain (0.78) and neck pain (0.81), whereas higher errors were observed in fibromyalgia (1.12) and migraine (0.90).

### 3.8. SHAP-Based Interpretation of Model Predictions

**Figure 5:**
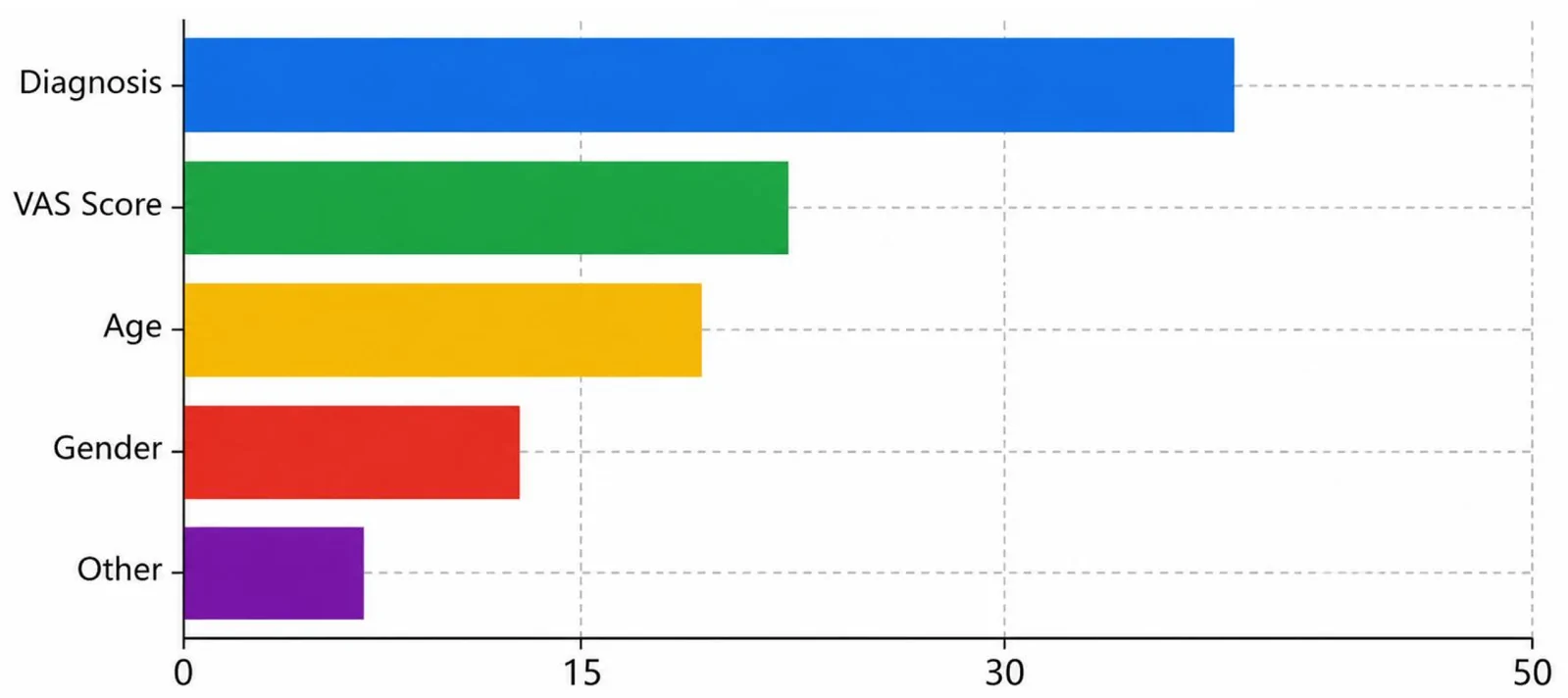
Feature Importance Analysis.

Feature importance was evaluated using SHAP (SHapley Additive exPlanations) summary values, normalized to percentage contributions to reflect global feature impacts on model predictions. Within the retrospective outcome-prediction analysis, feature importance analysis using SHAP values indicated that diagnostic category was the most influential predictor of clinical improvement (31.2%), followed by treatment modality (27.6%) and number of treatment sessions (21.3%). These treatment-related variables—available only in the retrospective clinical records for outcome analysis—reflect observed treatment patterns and were not provided to the GETAIA recommendation module as baseline inputs at T0. Baseline VAS pain score, age, sex, symptom duration, and comorbidity status contributed to the model to varying degrees (Figure 5). These patterns represent model-derived associations within the retrospective dataset rather than causal effects or independently validated clinical determinants.

### 3.9. Prospective Pilot Feasibility and Preliminary Workflow Evaluation

In the prospective pilot evaluation (*n* = 42), GETAIA was incorporated into the point-of-care workflow, providing preliminary evidence of operational feasibility. Clinically meaningful improvement (≥3-point reduction in VAS score) was observed in 85.7% (18/21) of patients in the AI-assisted group compared with 71.4% (15/21) in the standard care group (*p* = 0.449; Mean VAS reduction: 3.9 ± 1.2 vs. 3.2 ± 1.4, *p* = 0.092; Cohen’s d = 0.54, 95% CI: −0.08 to 1.15). Given the non-randomized pragmatic allocation, small sample size (*n* = 42), and absence of blinding, these between-group differences should be interpreted as exploratory observations rather than evidence of clinical effectiveness or superiority. The primary purpose of the pilot was to assess point-of-care workflow integration and operational feasibility of the decision-support prototype.

### 3.10. Patient Satisfaction

Patient satisfaction was assessed at the completion of treatment using a standardized 5-point Likert Scale questionnaire (1 = Very Dissatisfied, 2 = Dissatisfied, 3 = Neutral, 4 = Satisfied, 5 = Very Satisfied; Cronbach’s alpha = 0.86). Overall satisfaction rates were operationalized as the proportion of patients reporting scores of 4 (“Satisfied”) or 5 (“Very Satisfied”). Satisfied patient proportions were highest in the cupping therapy group (85.7%), followed by acupuncture (78.5%) and mesotherapy (72.8%). A strong, statistically significant positive correlation was observed between the magnitude of pain reduction (VAS score decrease) and overall patient satisfaction (r = 0.74, *p* < 0.001).

### 3.11. Internal Model Fidelity and Preliminary Workflow Integration

**Figure 6:**
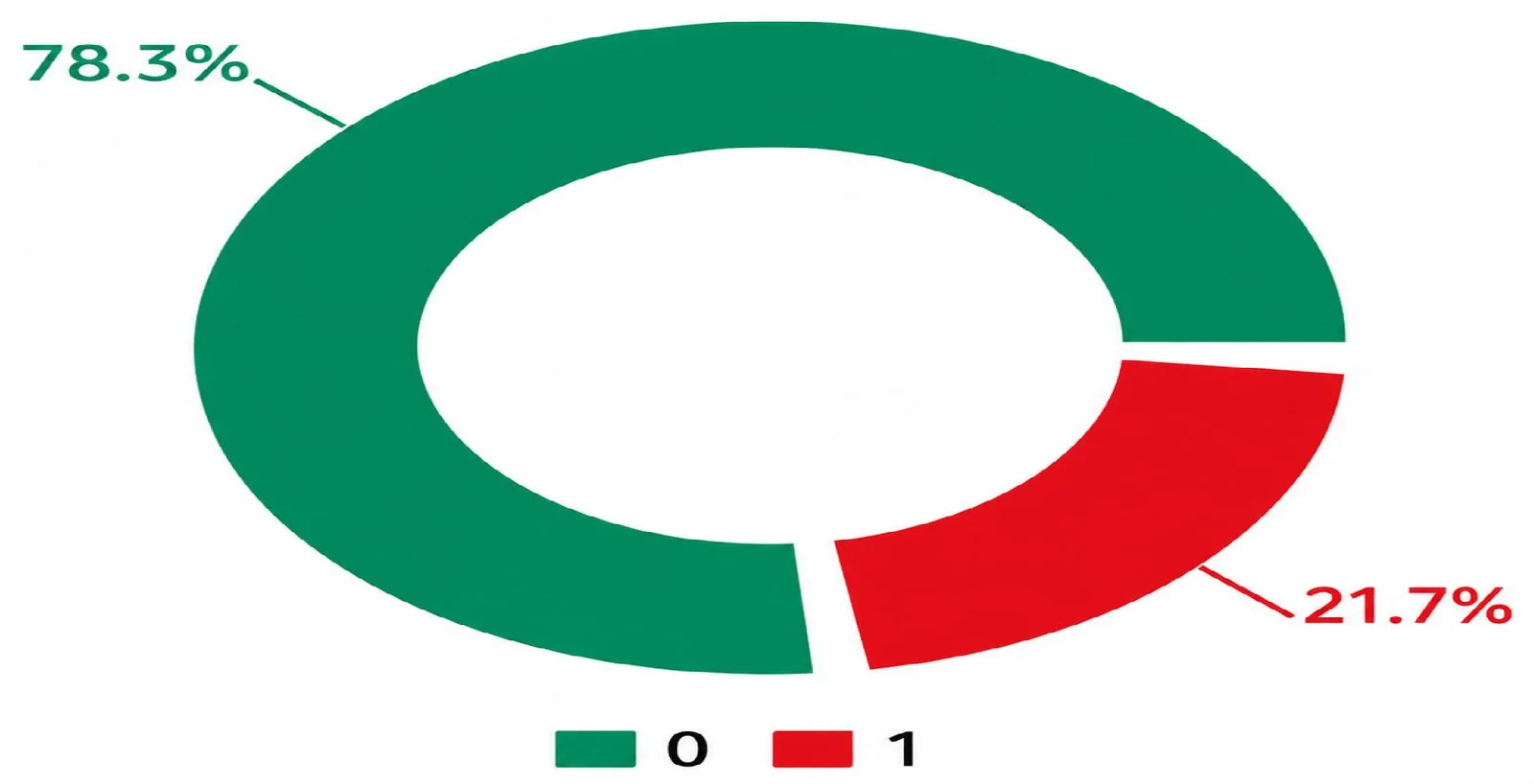
Internal Concordance Between AI Recommendations and Historical Clinician Treatment Decisions.

Concordance was assessed by comparing the modality recommended by GETAIA with the corresponding treatment modality recorded in the historical clinical record for the same patient. The GETAIA prototype demonstrated 78.3% concordance between its model-derived T&CM modality recommendations and the treatment modalities documented in the historical clinical records (Figure 6). This concordance analysis evaluated whether the modality recommended by the prototype corresponded to the modality documented in the historical record; it did not represent a separate clinical outcome-prediction model or an assessment of treatment effectiveness. This concordance was interpreted as an indicator of internal model fidelity, reflecting the extent to which the model reproduced treatment-selection patterns embedded in the retrospective clinical data. Because the model was developed using historical clinician decisions and treatment outcomes from the same clinical setting, this concordance may partly reflect the reproduction of pre-existing clinical decision patterns rather than an independent contribution of the model to clinical decision-making. Accordingly, this finding does not constitute evidence of improved clinical decision-making, treatment superiority, clinical effectiveness, or external validity. During the preliminary prospective pilot, the prototype was incorporated into the point-of-care workflow as a supportive decision tool to assess operational feasibility.

## 4. Discussion

This study developed and preliminarily evaluated a machine learning-based decision-support framework and GETAIA prototype within an integrative nursing context for personalized musculoskeletal pain management in a Traditional and Complementary Medicine (T&CM) setting. The study primarily involved retrospective development and evaluation of predictive models using clinical data from 487 patients, followed by integration of the models into a prototype workflow and a small prospective feasibility pilot. The retrospective models demonstrated good predictive performance on the held-out test dataset, whereas the prospective pilot provided preliminary information regarding point-of-care workflow integration and operational feasibility. However, these findings should be interpreted as evidence of model performance and early-stage prototype feasibility rather than evidence of a fully implemented or clinically validated decision support system. The non-randomized pilot was not designed or powered to establish clinical effectiveness, superiority over standard care, or readiness for routine clinical implementation. Accordingly, the findings provide a basis for further prospective evaluation rather than evidence supporting routine clinical deployment.

The prospective pilot should be interpreted primarily as a feasibility assessment rather than an evaluation of clinical effectiveness. The observed differences in pain-related outcomes between the AI-assisted and standard-care groups were exploratory and should be interpreted cautiously given the small sample size, non-randomized allocation, and absence of blinding. The pilot therefore provides preliminary information regarding the integration of GETAIA into point-of-care clinical workflow rather than robust evidence of comparative treatment effectiveness. Future adequately powered randomized studies are needed to determine whether the observed numerical trends translate into clinically meaningful benefits.

Musculoskeletal pain is among the leading causes of disability, functional impairment, and reduced quality of life worldwide and requires multidimensional and person-centered management strategies rather than exclusively biomedical approaches. Integrative nursing addresses the physical, psychological, and social dimensions of chronic pain through holistic assessment, symptom management, therapeutic relationships, and nonpharmacological care approaches [5]. In the present study, the significant pain reduction observed in the cupping therapy, acupuncture, and mesotherapy groups is consistent with previous evidence indicating that integrative and complementary interventions may contribute to symptom control and functional recovery in chronic musculoskeletal conditions [17]. These findings further support the relevance of integrative nursing models for individualized, patient-centered pain management and health promotion.

Rather than merely reflecting standalone modality efficacy, the differential treatment responses observed across subgroups highlight the high predictive weight of baseline anatomical location, diagnosis type, and pain severity within the decision-support architecture. Mechanistically, the strong responsiveness of axial musculoskeletal pain (low back and neck) to cupping therapy aligns with documented physiological pathways involving localized microcirculation enhancement, heme oxygenase-1 induction, and spinal neuromodulation [18,19]. Similarly, the observed alignment of headache/migraine with acupuncture and knee osteoarthritis with mesotherapy is consistent with the model capturing associations between diagnostic phenotypes and observed treatment patterns, rather than demonstrating specific biological or tissue-level treatment mechanisms [20,21]. Feature importance analysis further indicated that diagnostic category, baseline pain severity, treatment modality, and number of sessions contributed substantially to model predictions. These findings suggest that diagnostic phenotypes and baseline symptom profiles may be useful considerations when developing personalized decision-support approaches for integrative nursing care.

One of the main findings of this study was the good predictive performance of the machine learning models evaluated using the retrospective dataset. The primary XGBoost model demonstrated good classification performance on the held-out retrospective test set. The concordance analysis was considered separately as an internal model-fidelity assessment, indicating the extent to which the prototype reproduced historical clinician treatment-selection patterns. Because the model was trained on historical clinical decisions from the same dataset, this concordance should not be interpreted as evidence of improved clinical decision-making, clinical superiority, or external validity. The predictive modeling findings are consistent with current evidence suggesting that AI-based systems may assist clinicians in decision-making processes and support personalized care planning [10,11]. Importantly, AI integration in this study extended beyond diagnostic support and was embedded within nursing-oriented care processes, supporting standardized yet individualized health service delivery. While most existing nursing clinical decision support systems focus on acute care workflows, risk stratification, and protocol adherence, few have been designed to support individualized decision-making across multiple complementary and traditional medicine modalities within integrative nursing practice. The GETAIA prototype was designed to address this gap by integrating multimodal predictive analytics with selected clinical and nursing-relevant assessment parameters to facilitate personalized T&CM decision-making at the point of care [22,23].

From a nursing perspective, AI-supported systems may reduce variability in clinical decision-making, particularly in complementary and integrative care settings. Clinical decision-making processes in such settings are often heterogeneous and influenced by practitioner experience, which may affect consistency in care quality. In contrast, integrative nursing emphasizes continuous assessment of symptom burden, patient preferences, functional status, and treatment response. In this context, AI-supported systems may strengthen evidence-informed decision-making while remaining conceptually aligned with principles of respectful and inclusive care. However, the effects of GETAIA on these dimensions were not directly evaluated in the present study. Nevertheless, AI should be considered a supportive tool that enhances rather than replaces clinical nursing judgment. Therapeutic communication, holistic assessment, ethical sensitivity, and relational nursing care remain central components of effective integrative pain management and clinical skills education.

The findings also emphasize the importance of personalized pain management in integrative nursing practice. Diagnosis type, baseline pain severity, and the number of treatment sessions were associated with clinical improvement in the retrospective cohort, whereas concordance between AI recommendations and historical treatment decisions was evaluated separately as an internal model-fidelity measure rather than as evidence of clinical benefit. These retrospective associations are consistent with studies indicating that individualized treatment matching may be associated with favorable symptom outcomes [24,25]. The observed association between the number of treatment sessions and clinical improvement further highlights the importance of patient engagement and continuity of care, which are central principles of health promotion and integrative nursing.

Another feature of the prototype was the incorporation of explainable artificial intelligence (XAI) mechanisms. Ethical and patient-centered nursing care requires clinicians to understand and communicate the rationale underlying treatment recommendations. Feature importance analysis demonstrated that diagnosis, baseline pain severity, treatment modality, and session number were the most influential predictors in model outputs. The integration of XAI modules may support clinician trust, facilitate interdisciplinary communication, and contribute to ethically grounded health service delivery. Recent evidence indicates that explainability, interpretability, and usability are important considerations for the safe and effective integration of AI into clinical decision support systems [26,27,28].

The relatively high predictive contributions of diagnostic category, baseline pain severity, treatment modality, and number of sessions indicate that these variables were important within the retrospective prediction framework. These associations should be interpreted as model-derived patterns rather than evidence of causal treatment mechanisms or independently validated determinants of therapeutic response. In particular, the concordance analysis should be distinguished from these predictive associations because it primarily reflects the model’s ability to reproduce treatment-selection patterns observed in the historical dataset.

Statistical significance alone does not establish clinical utility or effectiveness. In the present study, the statistically significant findings observed in the retrospective cohort describe associations and within-group changes and should not be conflated with evidence from the prospective feasibility pilot. The pilot was not designed or powered to establish comparative clinical effectiveness, and its between-group findings were exploratory. In the retrospective dataset, the observed treatment-associated changes included substantial effect sizes (Cohen’s d = 1.05 for cupping therapy; d = 0.82 for acupuncture) and meaningful absolute outcome changes (a mean VAS score reduction exceeding 3.0 points). Furthermore, probability calibration analysis (Brier Score = 0.12) indicated favorable agreement between predicted probabilities and observed outcomes within the retrospective test dataset. However, this calibration performance should be interpreted as an internal model evaluation rather than evidence of validated clinical decision thresholds or a justification for relying on uncalibrated confidence scores, particularly because uncertainty estimation is increasingly recognized as an important component of trustworthy AI-based clinical decision support [29].

Regarding outcome measurement, the selection of a ≥3-point VAS drop as a binary responder criterion provided a conservative and clinically meaningful threshold aligned with validated Minimal Clinically Important Difference (MCID) standards in musculoskeletal pain research [15,16]. Although baseline pain severity inherently affects absolute drop magnitude—as reflected by baseline VAS ranking among the top model predictors—adjusting for initial severity in multivariable models ensured that clinical success classification remained robust across diagnostic subgroups.

The mobile-based AI-supported decision-support prototype may have potential utility for supporting nursing workflow and accessibility within integrative care services. Clinical decision support systems integrated into mobile health technologies may facilitate rapid symptom assessment, individualized intervention planning, and longitudinal monitoring of treatment response [30]. Such systems may be particularly valuable in rehabilitation and community-based care settings by supporting continuity of care. Additionally, AI-supported systems may enhance clinical skills education by providing structured, data-driven feedback and improving consistency in clinical reasoning among less experienced clinicians.

### 4.1. The Role of Integrative Nursing and Nursing-Sensitive Outcomes

To frame the GETAIA decision-support ecosystem within integrative nursing practice, the clinical workflow delineates complementary yet distinct scopes of practice between physicians and nursing staff. While formal medical diagnosis and therapy prescription remain under physician authority, integrative nurses play a pivotal role in baseline clinical evaluation, comprehensive symptom screening, baseline Visual Analog Scale (VAS) pain scoring, and holistic patient assessment.

The decision-support prototype is intended to support nursing staff during triage, patient counseling, symptom monitoring, and supportive care planning. Nurses utilize the system’s predictive insights to guide patient education, manage expectations regarding non-pharmacological T&CM modalities, and optimize procedural preparation. Furthermore, our findings highlight patient-centered outcomes relevant to nursing care, including the number of treatment sessions, pain outcomes, and overall patient satisfaction. These outcomes may be influenced by nursing assessment, patient education, therapeutic communication, and continuity of care [31]. In this context, the integration of AI-supported decision tools may assist nurses in identifying patient-specific needs, monitoring treatment responses, and delivering more consistent and individualized supportive care.

### 4.2. Limitations and Future Research

Several limitations of this study should be acknowledged. First, while the machine learning models demonstrated high predictive performance on historical data, the AI-Assisted Clinical Decision Support System framework presented here represents a preliminary decision-support prototype rather than a fully deployed enterprise system. Second, a key methodological limitation relates to the retrospective concordance analysis (Section 3.11); because the model was trained on historical clinician decisions and treatment outcomes, the observed concordance primarily reflects the model’s fidelity in reproducing historical clinical decision patterns rather than improved decision-making, clinical effectiveness, treatment superiority, or external validity. Third, the study was primarily conducted using retrospective data from a single T&CM center, which may limit the external validity and generalizability of the findings. Fourth, reliance on self-reported Visual Analog Scale (VAS) scores may introduce measurement subjectivity. Fifth, model performance varied across diagnostic subgroups—with lower predictive accuracy observed in fibromyalgia cases—highlighting the need for larger, more balanced datasets. Sixth, although a point-of-care pilot evaluation was conducted with 42 patients, the preliminary sample size, non-randomized sequential allocation, and pragmatic unblinded design require cautious interpretation. These pilot findings provide preliminary evidence of workflow integration and operational feasibility rather than evidence of unconfounded therapeutic effectiveness or superiority. Finally, broader ethical, legal, and governance challenges surrounding AI integration in nursing practice require ongoing investigation.

An additional limitation concerns the operationalization of the biopsychosocial framework. Although the conceptual framework of GETAIA is informed by the biopsychosocial nature of chronic pain, the variables available in the retrospective dataset provided only a limited and indirect representation of psychological and social/contextual domains. Baseline VAS pain intensity and symptom duration do not directly measure psychological distress or emotional burden, while demographic characteristics, treatment utilization, session frequency, and patient satisfaction do not comprehensively capture social support, family environment, socioeconomic circumstances, cultural factors, or caregiving relationships. No standardized psychometric or psychosocial instruments were included in the present dataset. Therefore, the current findings should not be interpreted as demonstrating a comprehensive biopsychosocial assessment. Future prospective studies should incorporate validated psychological and social measures to enable a more complete evaluation of the biopsychosocial dimensions of chronic pain and to strengthen the personalization capacity of the GETAIA framework.

Future research should focus on prospective, multicenter randomized controlled trials (RCTs) with larger and more diverse patient populations to comprehensively evaluate real-time clinical integration, workflow impact, clinical effectiveness, and long-term outcomes. In addition, integration of wearable technologies, real-time physiological monitoring, and longitudinal patient-reported outcomes (PROMs) may further strengthen evidence-informed integrative nursing strategies. Qualitative studies exploring patient and clinician user experiences with the GETAIA mobile platform will also enhance understanding of respectful healthcare implementation. Finally, further development of transparent and explainable AI (XAI) mechanisms will be essential for building clinician trust, ensuring safety, and promoting interdisciplinary collaboration in clinical practice. Accordingly, the present findings should be interpreted as evidence of model performance and preliminary prototype feasibility rather than evidence of the effectiveness of a fully implemented clinical decision support system in routine practice.

### 4.3. Mapping Study Variables to the Biopsychosocial Framework

To clarify the conceptual relationship between the GETAIA architecture and the biopsychosocial framework, the variables available in the retrospective dataset were considered in relation to biological, psychological/perceptual, and social/contextual aspects of chronic musculoskeletal pain. However, these variables should be regarded as a pragmatic and limited representation of these domains rather than as a comprehensive biopsychosocial assessment.

- Biological Dimension: The biological component was represented primarily by clinical diagnoses, anatomical pain distribution, baseline pain intensity (VAS scores), and comorbidity status.
- Psychological/Perceptual Dimension: Baseline VAS pain scores provide information regarding the patient’s subjective perception of pain, while symptom duration provides information about pain chronicity. However, neither variable constitutes a direct measure of psychological distress, emotional burden, anxiety, depression, coping, or other psychological processes associated with chronic pain.
- Social and Contextual Dimension: Demographic characteristics such as age and sex, together with treatment-related variables and patient satisfaction, provide limited contextual information relevant to care utilization and patient experience. These variables do not comprehensively capture social support, family environment, socioeconomic circumstances, cultural factors, or therapeutic and caregiving relationships.

Accordingly, the variables included in the present study should not be interpreted as a comprehensive operationalization of the biopsychosocial model. Rather, they represent the clinical and contextual information that was available within the retrospective dataset and could be incorporated into the preliminary decision-support framework. Standardized psychosocial and psychometric measures were not included in the present dataset. Future prospective iterations of GETAIA should incorporate validated measures of psychological distress, coping, social support, quality of life, and other relevant psychosocial determinants to strengthen its biopsychosocial personalization capacity.

### 4.4. Implications for Nursing Practice

The GETAIA prototype may provide a basis for future investigation by supporting individualized symptom assessment, continuity of care, and evidence-informed pain management. Explainable AI mechanisms are intended to support clinician understanding and interdisciplinary communication while preserving the therapeutic and holistic foundations of nursing care. In integrative and complementary care settings, these systems may provide a structured framework for standardized and individualized clinical decision support and may support the development of advanced clinical skills in modern nursing practice. Their potential contribution to inclusive and respectful healthcare delivery remains to be evaluated in future prospective studies.

## 5. Conclusions

This study presents the development and preliminary evaluation of a machine learning-based decision-support framework and GETAIA prototype for personalized musculoskeletal pain management within an integrative nursing framework in T&CM settings. In the retrospective component, the evaluated machine learning models demonstrated good predictive performance on a held-out test dataset, while cupping therapy, acupuncture, and mesotherapy were associated with significant reductions in pain intensity. The prototype demonstrated 78.3% concordance between model-derived T&CM modality recommendations and historical clinician treatment decisions; however, this concordance represents an internal model-fidelity measure and should not be interpreted as evidence of clinical validation, improved decision-making, treatment superiority, or external validity. The prospective pilot provided preliminary observations regarding point-of-care workflow integration, with favorable numerical differences in the AI-assisted group that were not statistically significant. These findings should therefore be interpreted as exploratory feasibility observations rather than evidence of clinical effectiveness.

The present study does not establish the effectiveness, superiority, routine-care readiness, or external validity of a fully implemented clinical decision support system. Rather, it provides an initial empirical basis for further investigation of the machine learning framework and the feasibility of the GETAIA prototype. Explainable AI and mobile integration were incorporated as design features intended to support transparency and point-of-care decision support; however, their effects on clinician trust, patient outcomes, workflow efficiency, accessibility, or continuity of care were not directly evaluated in the present study. AI should therefore be regarded as a supportive tool that complements, rather than replaces, professional clinical judgment, while holistic assessment, therapeutic relationships, and individualized nursing care remain central to integrative pain management. Future studies should employ larger, prospective, multicenter designs with independent external validation and appropriately designed comparative evaluations to determine clinical effectiveness, usability, workflow impact, generalizability, and implementation readiness before consideration of routine clinical deployment.

## Figures and Tables

**Table 1:** Sociodemographic and Clinical Characteristics of the Study Sample (*n* = 487).

Variable	Category	*n* (%)/Mean ± SD
Age (years)		48.2 ± 14.7
Gender	Female	307 (63.0)
	Male	180 (37.0)
Treatment Group	Acupuncture	205 (42.1)
	Cupping therapy	168 (34.5)
	Mesotherapy	114 (23.4)

Data are presented as mean ± standard deviation (SD) or *n* (%), as appropriate.

## Data Availability

The datasets generated and/or analyzed during the current study are not publicly available due to patient confidentiality and privacy restrictions. However, data are available from the corresponding author upon reasonable request.

## Abbreviations

The following abbreviations are used in this manuscript:

Al	Artificial Intelligence
T&CM	Traditional and Complementary Medicine
VAS	Visual Analog Scale
MAE	Mean Absolute Error
OR	Odds Ratio
CI	Confidence Interval
XAI	Explainable Artificial Intelligence
SD	Standard Deviation
